# Febuxostat ameliorates secondary progressive experimental autoimmune encephalomyelitis by restoring mitochondrial energy production in a GOT2-dependent manner

**DOI:** 10.1371/journal.pone.0187215

**Published:** 2017-11-06

**Authors:** Josephe A. Honorat, Yuji Nakatsuji, Mikito Shimizu, Makoto Kinoshita, Hisae Sumi-Akamaru, Tsutomu Sasaki, Kazushiro Takata, Toru Koda, Akiko Namba, Kazuya Yamashita, Eri Sanda, Manabu Sakaguchi, Atsushi Kumanogoh, Takashi Shirakura, Mizuho Tamura, Saburo Sakoda, Hideki Mochizuki, Tatsusada Okuno

**Affiliations:** 1 Department of Neurology, Osaka University Graduate School of Medicine, Suita, Osaka, Japan; 2 Department of Neurology, Osaka General Medical Center, Osaka, Osaka, Japan; 3 Department of Respiratory Medicine, Allergy and Rheumatic Diseases, Osaka University Graduate School of Medicine, Suita, Osaka, Japan; 4 Pharmaceutical Development Research Laboratories, Teijin Pharma Ltd., Hino, Tokyo, Japan; 5 Department of Neurology, National Hospital Organization Toneyama National Hospital, Toyonaka, Osaka, Japan; Nagoya Daigaku, JAPAN

## Abstract

Oxidative stress and mitochondrial dysfunction are important determinants of neurodegeneration in secondary progressive multiple sclerosis (SPMS). We previously showed that febuxostat, a xanthine oxidase inhibitor, ameliorated both relapsing-remitting and secondary progressive experimental autoimmune encephalomyelitis (EAE) by preventing neurodegeneration in mice. In this study, we investigated how febuxostat protects neuron in secondary progressive EAE. A DNA microarray analysis revealed that febuxostat treatment increased the CNS expression of several mitochondria-related genes in EAE mice, most notably including *GOT2*, which encodes glutamate oxaloacetate transaminase 2 (GOT2). GOT2 is a mitochondrial enzyme that oxidizes glutamate to produce α-ketoglutarate for the Krebs cycle, eventually leading to the production of adenosine triphosphate (ATP). Whereas GOT2 expression was decreased in the spinal cord during the chronic progressive phase of EAE, febuxostat-treated EAE mice showed increased GOT2 expression. Moreover, febuxostat treatment of Neuro2a cells *in vitro* ameliorated ATP exhaustion induced by rotenone application. The ability of febuxostat to preserve ATP production in the presence of rotenone was significantly reduced by GOT2 siRNA. GOT2-mediated ATP synthesis may be a pivotal mechanism underlying the protective effect of febuxostat against neurodegeneration in EAE. Accordingly, febuxostat may also have clinical utility as a disease-modifying drug in SPMS.

## Introduction

More than half of patients with relapsing-remitting (RR) MS develop secondary progressive multiple sclerosis (SPMS) 20 years after initial disease onset. Although a number of disease-modifying drugs (DMDs) have been identified for the treatment of RRMS, currently available DMDs are largely ineffective for the treatment of SPMS, with the exception of Ocrelizumab [1]. Various pathological features have been described in patients with SPMS, including neurodegeneration, cortical demyelination, and meningeal inflammation; of these, progressive disability is mainly attributed to neurodegeneration [2–5]. Several mechanisms have been proposed to underlie neurodegeneration in SPMS. Oxidative stress and mitochondrial disturbances have been heavily implicated in neurodegeneration [6]. Recently, Nikic et al. described a variant of neurodegeneration termed focal axonal degeneration (FAD) that begins with focal swelling and is reversible or can progress to axonal fragmentation. Reactive oxygen species (ROS) are thought to cause intra-neurodegeneration mitochondrial injury leading to FAD [7, 8].

Febuxostat is a non-purine selective xanthine oxidase (XO) inhibitor that is currently used for the treatment of gout. In addition to the inhibition of uric acid synthesis, febuxostat decreases XO-mediated ROS production and improves mitochondrial function [9, 10]. We previously showed that febuxostat treatment ameliorated relapsing-remitting and secondary progressive subtypes of murine experimental autoimmune encephalomyelitis (EAE) by inhibiting the excess production of ROS and reducing neurodegeneration [11]. Since neurodegeneration is closely linked to mitochondrial dysfunction, we next hypothesized that febuxostat might improve neurodegeneration in EAE by compensating for mitochondrial dysfunction in addition to its effects on ROS. In the present study, we used a microarray analysis to evaluate changes in gene expression in the central nervous system (CNS) of NOD mice in which EAE is induced with MOG_35-55_, (NOD-EAE) following treatment with febuxostat. NOD-EAE mice have been known to show a self–limited acute neurological peak approximately 20 days after immunization, followed by a phase of irreversible progressive neurological impairment, thus serving as a model of secondary progressive multiple sclerosis [12]. Thereafter, we examined the ability of febuxostat to promote oxaloacetate transaminase (GOT2) expression as a method of restoring ATP synthesis *in vitro*. Our data inform the mechanisms of action of febuxostat in EAE and identify febuxostat as a clinical candidate for the treatment of SP-MS.

## Methods

### EAE induction, treatment and DNA microarray

All methods were performed in accordance with the relevant guidelines and regulations. All experimental procedures were approved by the Animal Care and Use Committee of Osaka University Graduate School of Medicine (permit number: 25-079-005). EAE was induced in 8 weeks old female non-obese diabetic (NOD)/ShiJcl mice (CLEA Japan, Inc.). EAE was induced using a modified method we previously reported [11]. In brief, NOD mice were subcutaneously immunized into both flanks with 100 μg MOG _35–55_ (myelin oligodendrocyte glycoprotein, MEVGWYRSPFSRVVHLYRNGK) emulsified in CFA (200 μl) containing *Mycobacterium tuberculosis* H37Ra (200 μg, Difco Laboratories), followed by intraperitoneal injections of 200 ng pertussis toxin (List Laboratories) on days 1 and 2. For treatment, Febuxostat (n = 8, 0.75 mg/kg/day; Teijin Pharma Ltd.) or Vehicle (n = 10, DMSO) in the drinking water was administered from 20 days post-immunization until the end of the study as previously described [11]. For the microarray analysis, brains and spinal cords were harvested at day 40 post-immunization and RNA was isolated and analyzed (Takara Biosciences). Forty-three mice used in this study were housed in microisolator cages at a modified pathogen-free barrier facility in the Animal Resource Center for Infectious Diseases, Research Institute for Microbial Diseases, Osaka University. All of the experimental procedures were performed following our institutional guidelines. Mice had free access to food and water ad libitum, and sodium pentobarbital anesthesia was applied in all of the surgery performed. All necessary steps were taken to ameliorate suffering to animals involved in our study, and mice were euthanized by CO_2_ inhalation. None of the mice in our study reached the criteria of defined humane endpoints. Mortality outside our planned euthanasia or humane endpoints neither occurred.

### RT-qPCR

cDNA was prepared from total RNA isolated from mice lumbar cords at different days post-immunization as previously described [11] and used for real-time PCR. The expression levels of GOT2 and β-actin or GAPDH as internal housekeeping controls were quantified using the following specific primers (Sigma-Aldrich): mGOT2 forward (5′ GGCTGACCAAGGAGTTCTCG), reverse (5′TCTGTTCCTTTGCACCTGGG); β-actin forward (5′ GATGACCCAGATCATGTTTGA), reverse (5′ GGAGCATAGCCCTCGTAG); GAPDH forward (5′ AATCCCATCACCATCTTCCA), reverse (5′ TGGACTCCACGACGTACTCA).

### Western blot analysis

Samples were lysed with CelLytic MT Mammalian Tissue Lysis Reagent (Sigma-Aldrich; USA) containing protease inhibitor cocktail (Thermo Fisher Scientific) and phosphatase inhibitor cocktail (Nacalai Tesque). Proteins were electrophoresed on 10% SDS-polyacrylamide gels and transferred onto nitrocellulose membranes (Bio-Rad Laboratories). Blots were incubated overnight at 4°C with one of the following primary antibodies: rabbit anti-GOT2 polyclonal antibody (1:100; Sigma-Aldrich) or mouse anti-β-actin monoclonal antibody (1:5000; Sigma-Aldrich). Blots were subsequently incubated with an appropriate horseradish peroxidase-conjugated secondary antibody (Goat anti-mouse IgG secondary antibody HRP; 1:2000; Invitrogen, Anti-Rabbit IgG HRP-linked antibody; 1:2000; CST) for 60 min and visualized using ECL Western Blotting Detection Reagent (GE Healthcare). Band images were captured and analyzed using an imaging system (Bio-RAD Laboratories).

### Immunohistochemistry

Fixed frozen tissue sections (10 μm) from lumbar spinal cords were incubated for 10 min with 1% H_2_O_2_ to quench endogenous peroxidase activity and then blocked with 2% bovine serum albumin (BSA) in phosphate-buffered saline (PBS) for 30 min. The primary antibodies used were a rabbit monoclonal antibody against GOT2 (1:50; Sigma-Aldrich) or mouse monoclonal antibody against GOT2 (1:100; Abcam) and then incubated over-night at 4°C. For diaminobenzidine staining, goat anti-rabbit IgG conjugated to peroxidase-labeled dextran polymer (Dako) was used as a secondary antibody. The sections were incubated for 30 minutes at room temperature with secondary antibody. Reaction products were visualized with 3, 3′-diaminobenzidine tetrahydrochloride (Vector Laboratories) and hematoxylin was used to counterstain cell nuclei. For double immunofluorescence, mouse anti-MAP2 monoclonal antibody (1:100; Sigma-Aldrich) or rabbit-anti TOM20 antibody (1:200; Santa Cruz) was used as a neuronal or a mitochondrial marker respectively. For immunocytochemistry, Neuro2A cells were fixed with 4% paraformaldehyde in PBS, blocked with 10% normal goat serum (NGS) and double-stained with mouse anti-GOT2 (1:100; Abcam) and rabbit anti-TOM20 (1:200; Santa Cruz) antibodies. Respectively, appropriate secondary antibody (anti-mouse IgG conjugated to Alexa 594 (1:500; abcam) and anti-rabbit IgG conjugated to Alexa 488 (1:500; abcam) were used.

### Cell culture and treatment

Neuro2a cells were grown in Dulbecco’s modified eagle’s medium (Sigma) containing 10% fetal bovine serum (FBS; Sigma) and 1% penicillin-streptomycin (Sigma). For GOT2 mRNA expression assays, cells were treated with febuxostat for 24 h and subsequently used for cDNA preparation, respectively. Alternatively, cells were stimulated with rotenone in the presence or absence of different concentrations of febuxostat for the indicated times.

### Small interfering RNA (siRNA) and ATP assay

siRNAs targeting GOT2 were purchased from Invitrogen. For RNA interference studies, Neuro2a cells were transfected with siRNAs using Lipofectamine siRNAmax (Invitrogen) according to manufacturer specifications. Forty-eight hours after transfection, cells were treated with febuxostat for 12 h and subsequently cells were treated with rotenone for 6 h. After treatment, cells were re-suspended and lysed for the assay of intracellular ATP content using an ATP Bioluminescence Assay Kit HS II (Roche).

### Statistical analysis

For EAE scores, significance among the groups was examined using nonparametric Mann-Whitney U test. One-way analysis of Student T-tests was used to compare two groups and a one-way analysis of variance (ANOVA) was used to compare more than two groups. The significance level was set at P ≤ 0.05.

## Results

### Febuxostat ameliorates SP-EAE

NOD mice were immunized with MOG_35-55_ to induce SP-EAE. SP-EAE mice developed acute attacks followed by remission and showed progressive chronic worsening of symptoms after day 20 post-immunization. SP-EAE mice treated with febuxostat (0.75 mg/kg body weight per day; Teijin Pharma Ltd., Tokyo, Japan) during the chronic progressive phase (from day 20 post-immunization) showed significant improvements in neurological disability compared to untreated SP-EAE mice (S1 Fig).

### Febuxostat increases GOT2 expression in EAE

**Fig 1:**
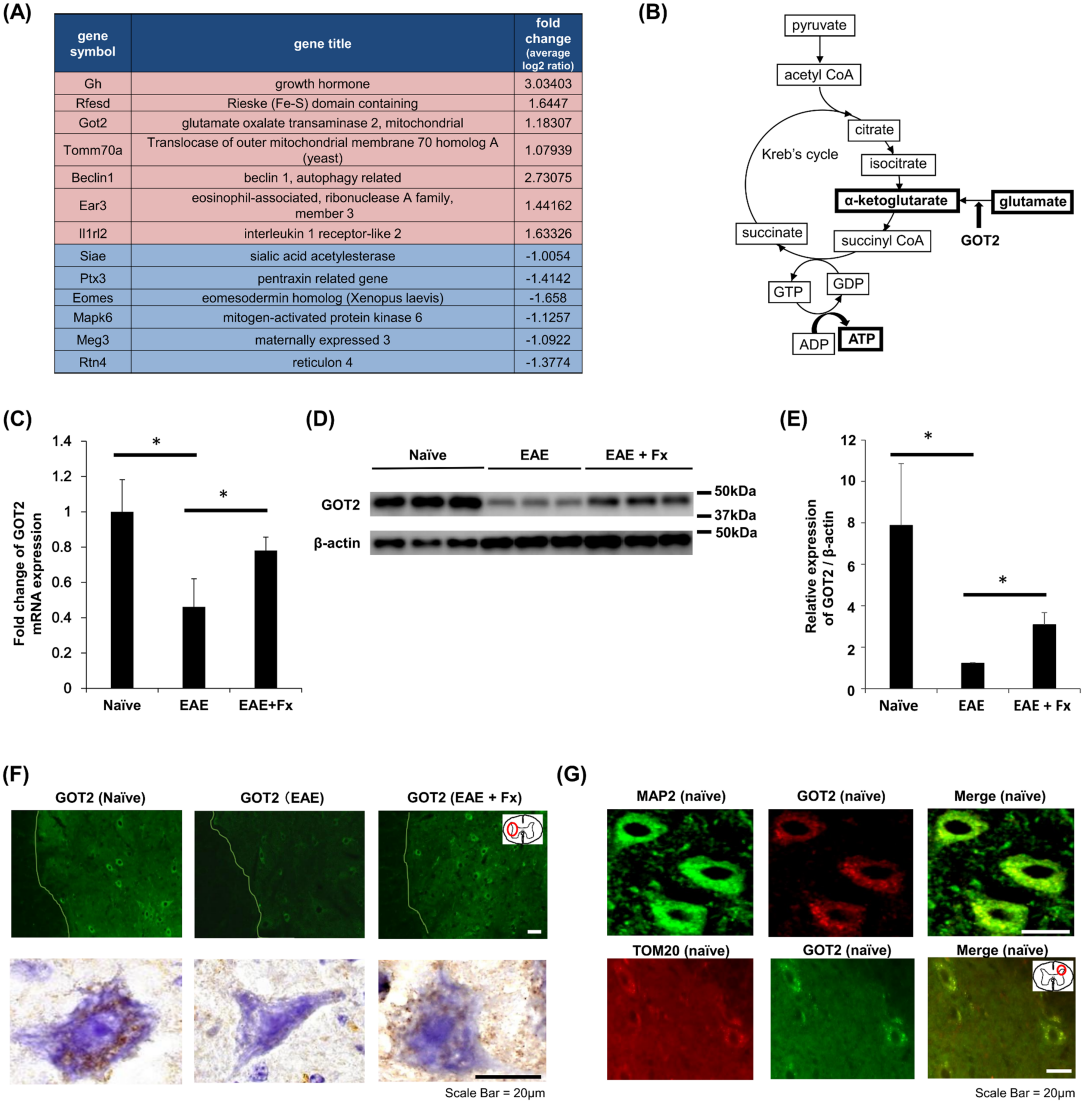
Febuxostat increases GOT2 expression in animal model of SPMS. (A) Representative alterations in gene expression detected by DNA microarray analysis in brains and spinal cords from mice with secondary progressive experimental autoimmune encephalomyelitis (SP-EAE) that were treated with febuxostat (Fx) or vehicle. Pink represents upregulation, while blue represents those downregulation of genes. (B) Schematic of an alternative pathway for ATP synthesis associated with GOT2 and the Krebs cycle. (C) RT-PCR analysis of GOT2 gene expression in spinal cords isolated from naïve, SP-EAE mice and SP-EAE mice treated with Fx (naïve; n = 3, EAE; n = 3, EAE+Fx; n = 3). (D) Western blot analysis of GOT2 protein expression in spinal cords isolated from naïve mice and SP-EAE mice treated with vehicle or Fx. β-actin was used as an internal control. (E) The relative expression level of GOT2 to β-actin for each depicted group (naïve, n = 3; SP-EAE, n = 3; SP-EAE+Fx, n = 3). (F) Immunohistochemical analysis of GOT2 expression in the lumbar cords isolated from naïve mice, SP-EAE mice or SP-EAE mice treated with Fx. Lines indicate borders between white matter and gray matter. Hematoxylin was used as a counterstain. The magnified image illustrates the dot-like expression of GOT2 in neuronal somata. (G) Immunofluorescent staining analysis of GOT2 and MAP2 or TOM20 in lumbar cords isolated from naïve mice indicates GOT2 is expressed in neuronal mitochondria.

In order to investigate the mechanism of neuroprotection conferred by febuxostat, a DNA microarray analysis was performed to compare CNS gene expression in febuxostat-treated and non-treated SP-EAE mice. The expression levels of various genes related to inflammation, cell cycle, and cellular transport were altered in febuxostat-treated versus non-treated SP-EAE mice; however, notably, several mitochondria-related genes were significantly up-regulated in febuxostat-treated compared to untreated SP-EAE mice. Altered targets were related to the import of mitochondrial precursor proteins (Tomm70a), autophagy (Beclin), the respiratory chain (Rfesd), and ATP synthesis (GOT2) (Fig 1A). GOT2 is an enzyme that exists in the inner membrane of mitochondria and converts glutamate to α-ketoglutarate in tandem with the transamination of oxaloacetate to aspartate and α-ketoglutarate; α-ketoglutarate then enters the Krebs cycle to produce ATP (Fig 1B). Because this pathway is an alternative route for ATP synthesis under conditions of high energy demand [13], we compared GOT2 gene expression in the spinal cords of naïve mice, SP-EAE mice and SP-EAE mice receiving febuxostat treatment using RT-qPCR and Western blotting. First, we confirmed enhanced gene expression of GOT2 in febuxostat-treated SP-EAE mice compared to control-treated SP-EAE mice (Fig 1C). Thereafter, a comparison of naïve and SP-EAE mice revealed that gene and protein expression levels of GOT2 were significantly decreased during the course of EAE, whereas febuxostat treatment restored GOT2 expression levels (Fig 1D and 1E).

**Fig 2:**
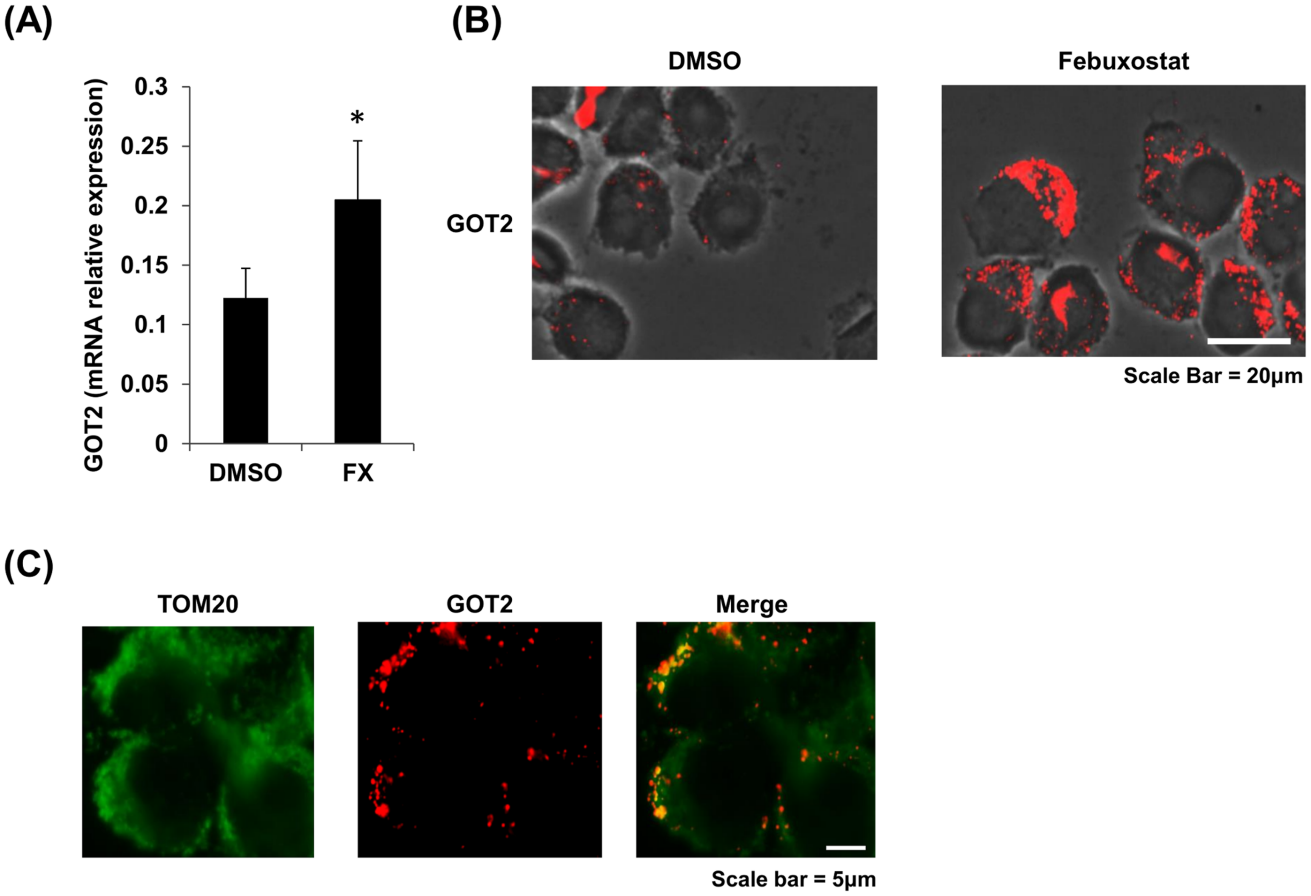
Febuxostat increases GOT2 expression in neural cell line. (A, B) Neuro2a cells were treated with 10 μM of Fx or vehicle (DMSO) for 24 h and the expression level of GOT2 was assessed by RT-PCR (A) and immunofluorescence (B). Data are mean ± SEM of triplicate cultures (A). * indicates P ≤ 0.05. One-way analysis of Student T-tests was used to compare two groups. (C) GOT2 (red) was colocalized with TOM20-positive mitochondria (green).

GOT2 up-regulation in the spinal cord of febuxostat-treated SP-EAE mice was also confirmed by immunohistochemistry (Fig 1F). A detailed morphological assessment of GOT2 distribution suggested that GOT2 was predominantly expressed in neuronal somata within gray matter of the spinal cord of naïve mice, SP-EAE mice and SP-EAE mice treated with febuxostat. Compared to untreated SP-EAE mice, febuxostat-treated SP-EAE mice showed increased GOT2 expression in neurons of gray matter lesions. To further confirm the localization of GOT2, we performed double immunofluorescence staining of GOT2 with neuronal marker Microtubule Associated Proteins 2 (MAP2) and mitochondrial marker TOM20, identifying that GOT2 was predominantly expressed in neuronal mitochondria (Fig 1G). By contrast, the expression of GOT2 in the white matter is low levels (Fig 1F). Consistent with these findings, both immunohistochemical and RT-PCR analyses demonstrated that 24-h febuxostat treatment led to enhanced expression of GOT2 in the mitochondria of Neuro2A cells (Fig 2A, 2B and 2C).

### Febuxostat rescues energy production in rotenone-treated Neuro2a cells

**Fig 3:**
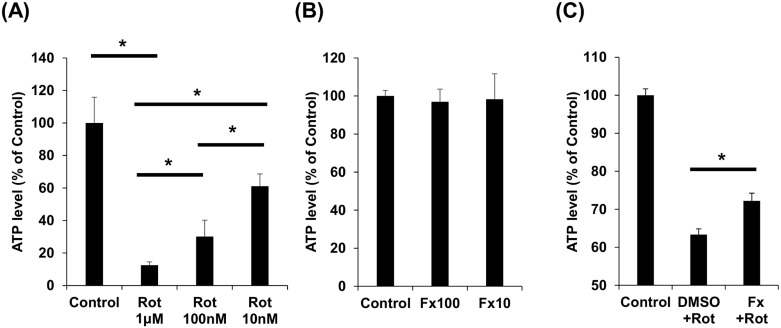
Febuxostat pretreatment increases ATP levels in Neuro2a cells incubated with rotenone. (A) Neuro2a cells were incubated with different concentrations of rotenone (Rot; 1 μM, 100 nM, 10 nM) for 6 h; then, cells were lysed and intracellular ATP was measured with a bioluminescence assay. (B) Neuro2a cells were treated with febuxostat (Fx; 100 μM, 10 μM) for 12 h and intracellular ATP was measured. (C) Neuro2a cells were pre-treated with febuxostat (10 μM) or vehicle (DMSO) for 12 h followed by incubation with rotenone (10 nM) for 6 h and intracellular ATP was measured. All experiments were performed in triplicate wells for each condition and repeated twice. The bars indicate amounts of ATP relative to the negative control. Error bars indicate standard error. * indicates P ≤ 0.05. One-way analysis of Student T-tests was used to compare two groups and a one-way analysis of variance (ANOVA) was used to compare more than two groups.

To examine the ability of febuxostat to rescue mitochondrial dysfunction in neurons, we investigated the ability of febuxostat to rescue ATP production in rotenone-treated Neuro2a cells. As expected, mitochondrial complex inhibition with rotenone treatment decreased steady-state ATP levels in a concentration-dependent manner (Fig 3A). Febuxostat alone did not affect steady-state ATP levels in Neuro2a cells (Fig 3B). Pretreatment with 10 μM febuxostat prior to rotenone treatment rescued ATP production such that levels were higher than those of the rotenone treated cells (Fig 3B and 3C).

### Febuxostat preserves neuronal ATP production through GOT2

**Fig 4:**
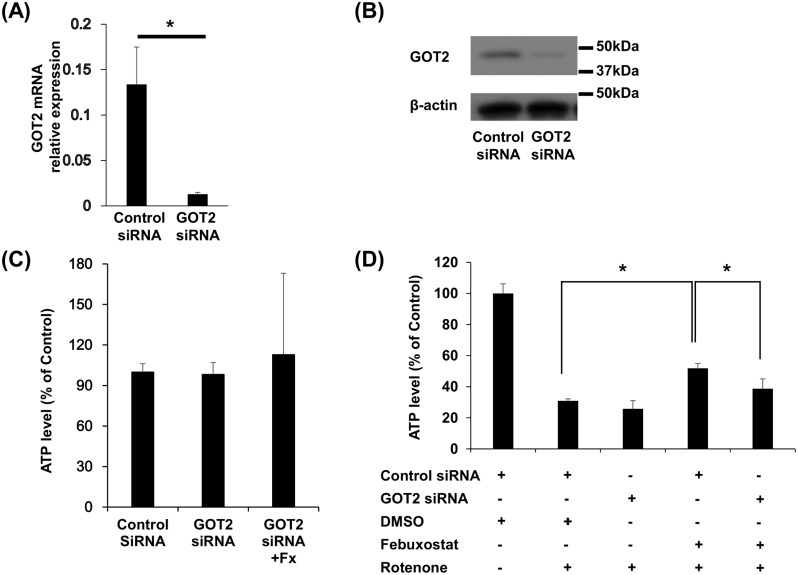
Febuxostat increases ATP production in mitochondrial dysfunction via GOT2. Neuro2a cells were transfected with control siRNA or GOT2 siRNA. RT-PCR analysis (A) and western blot analysis (B) confirmed the silencing of GOT2 gene and protein expression, respectively. (C) ATP production was assessed in Neuro2a cells that were transfected either with control or GOT2-specific siRNA and treated with DMSO or 10 μM of Fx for 12 h. (D) Neuro2a cells that were transfected with either control or GOT2-specific siRNA were stimulated with 10 nM of rotenone (Rt, 6 h) after pre-treatment with DMSO or 10 μM of Fx for 12 h. Data are mean ± SEM of triplicate cultures. Error bars indicate standard error. All data are representative of 3 independent experiments. * indicates P ≤ 0.05. One-way analysis of Student T-tests was used to compare two groups and a one-way analysis of variance (ANOVA) was used to compare more than two groups.

Finally, we investigated whether increases in GOT2 expression promote a compensatory pathway of ATP production after mitochondrial dysfunction in Neuro2a cells. GOT2 was knocked-down using siRNA and specific silencing of GOT2 expression was confirmed by RT-PCR and Western blot analyses (Fig 4A and 4B). Neither knockdown of GOT2 nor febuxostat treatment alone altered steady-state ATP levels in Neuro2a cells (Fig 4C); however, the beneficial effect of febuxostat on ATP production in the presence of rotenone was abrogated in GOT2-silenced cells (Fig 4D). These results highlighted the essential role of GOT2 for maintaining ATP production in neuronal cells with mitochondrial dysfunction.

## Discussion

Mitochondrial damage or dysfunction preceding neurodegeneration is recognized as a pivotal pathophysiological feature in the pathogenesis of SPMS [14–17]. Accordingly, recent data suggest that energy failure is a fundamental determinant of neurodegeneration in MS, where energy demand is increased in neural cells [18, 19]. Moreover, mitochondrial dysfunction has been associated with progressive neurodegeneration in MS patients [15]. Febuxostat is a XO inhibitor that has demonstrated protective mitochondrial effects in myocardial infarction [9, 10]. In this study, we showed that febuxostat also exerts mitochondrial protection in neurons; febuxostat treatment up-regulated several mitochondrial genes in the CNS of SP-EAE mice, including genes encoding Tomm70a, Beclin, and GOT2. Because these proteins are involved in the regulation of mitochondrial function, the elimination of dysfunctional mitochondria through mitophagy, and a pathway leading to ATP synthesis, respectively, it can be hypothesized that febuxostat ameliorates neuronal damage in EAE by promoting protective responses to mitochondrial dysfunction.

We identified GOT2 as an important molecule for febuxostat-mediated neuroprotection in SP-EAE. GOT2 enhances ATP synthesis by providing α-ketoglutarate as a substrate for the Krebs cycle and thus promotes a compensatory pathway of ATP synthesis in cases of cellular energy deficiency [13, 20, 21]. Consistent with this role, knockdown of GOT2 in Neuro2a cells did not alter steady-state ATP production *in vitro*; however, GOT2 knockdown abrogated the ability of febuxostat to restore ATP production in Neuro2a cells treated with a mitochondrial complex inhibitor. These results suggest important roles of GOT2 in serving as a compensatory ATP source where excess energy production is required, and in the beneficial effects of febuxostat observed in SP-EAE models.

GOT2 catalyzes the production of aspartate, a precursor of N-acetyl aspartate (NAA). Accumulating evidence suggest NAA play an important role in neuronal/axonal health and integrity, associated with ATP production [22]. Various reports showed a significant reduction of NAA in gray matter and in normal appearing white matter in SPMS compared to RRMS and controls [23–25]. Although cytoplasmic role of GOT2 in SPMS remains still unknown, these findings indicate a correlation between low levels of NAA and MS progression. We hypothesize that the decrease of NAA in SPMS might partly reflect the decrease of GOT2 expression in neuronal cells as shown in our study.

Interestingly, we found that GOT2 expression was significantly decreased in the neuronal cell bodies of SP-EAE mice compared to naïve mice. Indeed, decreased ATP production has been reported in correlation with decreased GOT2 expression and function in preclinical models of traumatic brain injury and neurodegenerative disease [26, 27]. ATP depletion within neuronal cells results in both inhibition of axonal transport system and dysfunction of ATP-dependent ion channels [28, 29]. Impaired functions of ion channels such as Na/K-ATPase lead to cytosolic Ca^2+^ overload and subsequent Ca^2+^-mediated axonal degradation [29]. Thus, the process of neurodegeneration might be accelerated by the insufficiency of energy supply due to decreased expression of GOT2 in neural cells. In line with these observations, the present data suggest that febuxostat potentiates the enhanced production of ATP by inducing the expression of neuronal GOT2, though the possibility remains that GOT2 expressed on glial cells and infiltrating inflammatory cells is involved in the effect of febuxostat.

We and others have reported the utility of ROS inhibitors in animal models of neurodegenerative disease, including in EAE [11, 30, 31]. Although the mechanism by which febuxostat promotes GOT2 expression and improves mitochondrial function remains unknown, previous report showed nuclear genes encoding mitochondrial protein including GOT2 is down–regulated under hypoxic state [32]. In addition, several lines of evidence support endothelial dysfunction related to xanthine oxidase activity [33]. Thus, one of the possibilities is that febuxostat treatment ameliorates hypoxic state of peripheral tissues by improving endothelial functions, resulting in the up-regulation of GOT2 gene expression. Alternative hypothesis includes direct effects of febuxostat on mitochondrial protein quality control in neuronal cells. However, the latter possibility awaits future investigation to be verified.

Taken together, febuxostat may be useful as a DMD for SPMS that acts by reducing ROS production and restoring mitochondrial energy supply during the course of inflammation and neurodegeneration.

## Supporting information

S1 Fig Febuxostat treatment ameliorates secondary progressive experimental autoimmune encephalomyelitis. Clinical scores in non-obese diabetic/ShiJcl mice treated with 0.75 mg/kg of febuxostat (open cycle, n = 8) or control vehicle (filled cycle, n = 10). Mice were treated with febuxostat or DMSO in drinking water from day 20 post-immunization until the end of the study. Data are mean ± SEM. * indicates P ≤ 0.05. Significance among the groups was examined using nonparametric Mann-Whitney U test.
(PDF)

S2 Fig The ARRIVE guideline checklist. (PDF)
