# Supplementary figures and images for: Febuxostat ameliorates secondary progressive experimental autoimmune encephalomyelitis by restoring mitochondrial energy production in a GOT2-dependent manner
Source: PLoS One. 2017 Nov 6;12(11):e0187215. doi: 10.1371/journal.pone.0187215 (PMC5673182; doi:10.1371/journal.pone.0187215)

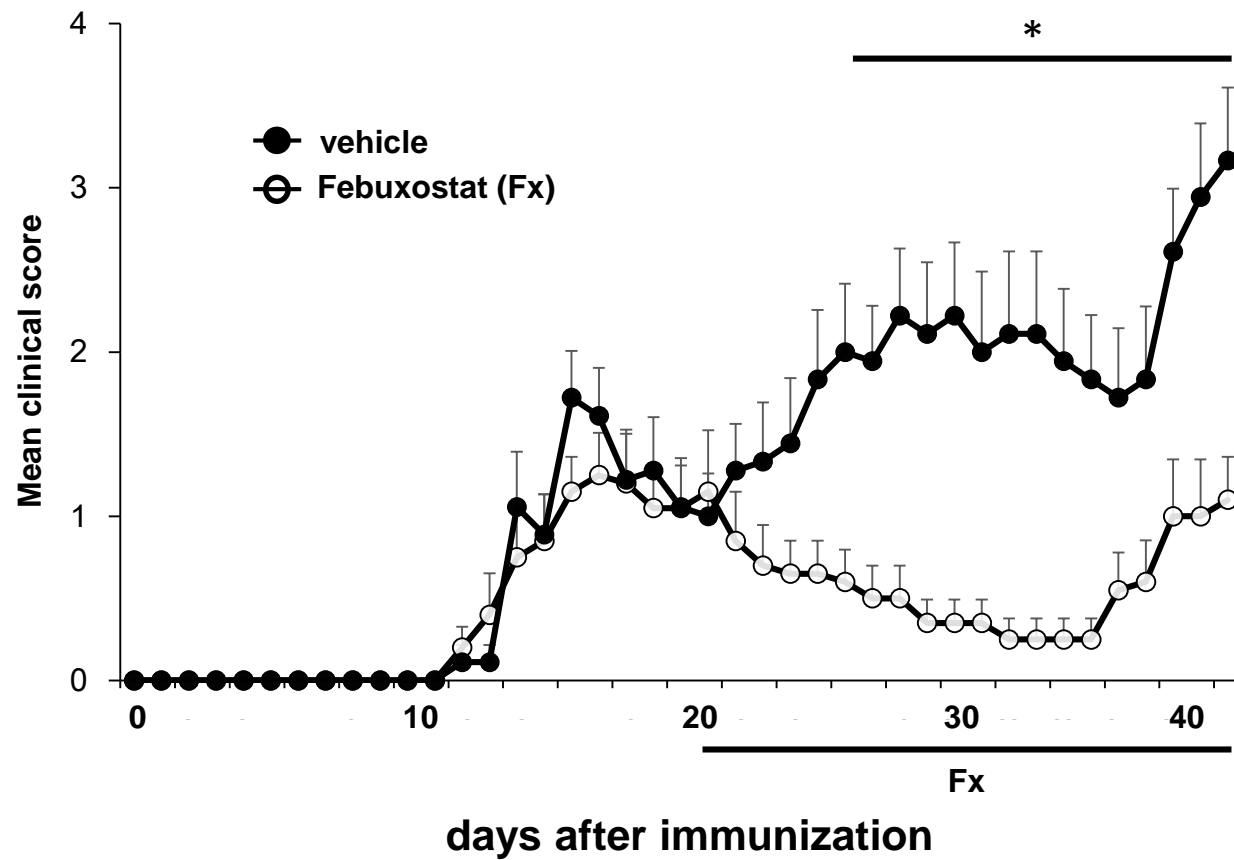

Supplement: S1 Fig — Clinical scores in non-obese diabetic/ShiJcl mice treated with 0.75 mg/kg of febuxostat (open cycle, n = 8) or control vehicle (filled cycle, n = 10). Mice were treated with febuxostat or DMSO in drinking water from day 20 post-immunization until the end of the study. Data are mean ± SEM. * indicates P ≤ 0.05. Significance among the groups was examined using nonparametric Mann-Whitney U test. (PDF) [file pone.0187215.s001.pdf]
